# Microwave-Assisted In-Situ Synthesis of Polyethersulfone–ZnO Nanocomposite Membranes for Dye Removal: Enhanced Antifouling, Self-Cleaning, and Antibacterial Properties

**DOI:** 10.3390/polym17030398

**Published:** 2025-02-02

**Authors:** Lassaad Gzara, Ibtissem Ounifi, Hussam Organji, Faïçal Khlissa, Iqbal Ahmed Moujdin, Abdulmohsen Omar Alsaiari, Mohamed Abdel Salam, Amor Hafiane

**Affiliations:** 1 Center of Excellence in Desalination Technology, King Abdulaziz University, P.O. Box 80200, Jeddah 21589, Saudi Arabia; haorganji@kau.edu.sa (H.O.); irajboot@kau.edu.sa (I.A.M.); aoalsaiari@kau.edu.sa (A.O.A.); 2 Laboratory, Water, Membrane and Environmental Biotechnology, Centre of Research and Water Technologies, Technopark of Borj-Cedria, BP 273, Soliman 8020, Tunisia; ibtissem.ounifi1988@gmail.com (I.O.); amor.hafiane@certe.rnrt.tn (A.H.); 3 Physical Chemistry Laboratory of Mineral Materials and Their Applications, National Center of Research in Materials Science, BP 73, Soliman 8027, Tunisia; fa.khlissa@cnrsrn.rnt.tn; 4 Department of Chemistry, Faculty of Science, King Abdulaziz University, P.O. Box 80200, Jeddah 21589, Saudi Arabia; masalam16@hotmail.com

**Keywords:** nanocomposite membrane, microwave-assisted synthesis, ZnO nanoparticle, dye removal

## Abstract

Microwave-assisted synthesis presents a promising method for enhancing the formation of nanocomposites due to its rapid heating and uniform energy distribution. In this study, we successfully fabricated polyethersulfone–zinc-oxide (PES-ZnO) nanocomposite membranes by exposing PES/ZnCl_2_/DMF dope solutions to microwave radiation. Before synthesizing the membranes, zinc-oxide nanoparticles (ZnO-NPs) were optimized in an organic phase using microwave radiation to ensure effective nanoparticle formation. The synthesis of ZnO-NPs in DMF solvent was validated through UV–Vis spectroscopy, X-ray diffraction (XRD), and Dynamic Light Scattering (DLS). We examined the surface morphology and roughness of the PES-ZnO membranes through Atomic Force Microscopy (AFM) and Scanning Electron Microscopy (SEM). Moreover, we assessed the membranes’ hydrophilicity, permeability, and physicochemical properties through contact-angle measurements, pure water flux tests, water uptake assessments, and porosity tests. Energy-dispersive X-ray spectroscopy (EDS) and X-ray diffraction (XRD) verified the successful integration of ZnO nanoparticles (ZnO-NPs) into the membrane matrix. The results indicate that including ZnO-NPs significantly improves the membrane’s permeability and hydrophilicity. The nanocomposite membranes exhibited high dye rejection efficiency, with ZnO-NPs facilitating photocatalytic self-cleaning properties. Antibacterial tests also demonstrated a substantial inhibition of common bacteria, suggesting enhanced resistance to biofouling. This research highlights the potential of microwave-assisted PES-ZnO nanocomposite membranes as effective and sustainable solutions for wastewater treatment, offering scalable applications along with added benefits of antifouling, self-cleaning, and antibacterial properties.

## 1. Introduction

The global water crisis is a pressing issue stemming from dwindling water resources, heightened demand for clean water, and the contamination of drinking water sources by both natural and man-made factors. Implementing water treatment is vital for industrial endeavors as it plays a key role in safeguarding the environment [1]. Although several techniques are available for treating water and wastewater, including electrochemistry, advanced oxidation, and adsorption [2,3,4], these methods have several drawbacks such as being expensive, using toxic substances, or being impractical for large-scale applications [5]. However, in recent years, membrane technology has significantly improved water treatment efficiency. It is commonly used to treat textile wastewater, desalinate seawater, remove heavy metals, and eliminate pathogenic bacteria [6]. Membrane processes offer several benefits, including simple operational control, low energy consumption, a compact design, and the capability to recover substances without the need for additional chemicals [7].

Various types of polymers are utilized in the fabrication of membranes, including cellulose acetate (CA) [8], polyvinylidene fluoride (PVDF) [9], and polyethersulfone (PES) [10]. PES stands out from these polymers due to its versatility, amorphous structure, exceptional performance, remarkable thermal properties, strong mechanical resistance, and high chemical stability. PES membranes are susceptible to membrane fouling due to their low hydrophilicity [11,12]. Fouling takes place when salts, macromolecules, and particles build up on the surface and within the membrane.

There are two types of fouling: (i) reversible fouling [13], caused by loosely bonded fouling that typically forms on the upper surface of the membrane, which can be removed by water cleaning; and (ii) irreversible fouling [14], caused by various compounds strongly binding with the membrane, which cannot be removed by physical cleaning.

Many works aim to modify the membrane properties and performances by reducing the fouling phenomenon through numerous additives [15]. Indeed, to form various PES-based nanocomposite membranes, PES was mixed with different nanomaterials, such as graphene oxide, TiO_2_ [16], SiO_2_ [17], Al_2_O_3_ [18], ZnO-NPs [19], Ag nanoparticles [20], and Co nanoparticles [21].

Zhao S et al. [22] produced composite membranes, PES-ZnO, and demonstrated that the modified membranes had a more porous structure, higher hydrophilicity, and improved thermal stability compared to pure PES membranes. Tutu et al. [23] developed PES-ZnO composite membranes with enhanced performance, including a 200% increase in flow rate, improved hydrophilicity, and enhanced antifouling properties. The integration of ZnO-NPs into the PES membrane matrix was observed to enhance hydrophilicity, promote pore formation, and result in significant improvements in flux and rejection rates [24]. However, the fouling resistance was only moderately improved compared to the original membrane. Jo et al. [25] introduced NH_2_ to the PES membrane and then applied ZnO on its surface to create a bond. Consequently, the modified membranes exhibited antibacterial properties against Escherichia coli and Staphylococcus aureus, unlike the unmodified ones.

So far, traditional methods for creating nanocomposite PES-ZnO membranes have involved incorporating ZnO-NPs into the polymeric solution or applying a surface coating. Microwave-assisted synthesis is an innovative and efficient method for creating nanocomposite membranes. It offers several advantages over conventional synthesis methods, such as faster reaction times, uniform heating, enhanced particle distribution, and the ability to control the morphology of the nanomaterials. In the realm of nanocomposite membranes, microwave synthesis is commonly used to integrate nanoparticles or nanomaterials into polymer matrices to improve properties like mechanical strength, permeability, and antifouling capabilities. The microwave-assisted synthesis of polyethersulfone (PES)–zinc-oxide (ZnO) nanocomposite membranes involves incorporating ZnO-NPs into a PES matrix. In this process, zinc chloride (ZnCl_2_) acts as the precursor for ZnO nanoparticle formation, and microwave irradiation enables rapid, uniform heating, resulting in the efficient in situ synthesis of ZnO-NPs within the PES polymer matrix. This method enhances the resulting membrane’s mechanical properties, hydrophilicity, and antimicrobial activity, making it suitable for water purification, wastewater treatment, and antifouling applications.

Despite several studies confirming the efficiency of PES-ZnO nanocomposite membranes, the main issue with these membranes is the leakage of ZnO-NPs [26]. To address this problem, we conducted experiments to investigate the impact of integrating the microwave technique into the synthesis process of PES-ZnO nanocomposite membranes. After preparing the nanocomposite membranes, we characterized and tested them to assess their properties, performance, and capacity to remove dyes.

## 2. Materials and Methods

### 2.1. Materials and Reagents

Polyethersulfone, specifically PES Veradel 3000P with an average molecular weight of 63 kDa, was obtained from Solvay Speciality Polymers Italy S.P.A (VIALE LOMBARDIA 20, BOLLATE, 20021, Milano, Italy). The N,N-Dimethylformamide (DMF) with a purity of at least 99.9%, glucose, Polyvinylpyrrolidone (PVP) with a molecular weight of 10 kDa, bovine serum albumin (BSA) (66.5 kDa) in powder form, sodium hydroxide (NaOH), methylene blue, and Congo red were all purchased from Sigma Aldrich, Burlington, MA, USA.

### 2.2. Microwave-Assisted Synthesis of Zinc-Oxide Nanoparticles

A Speedwave XPERT microwave synthesis reactor from Berghof was utilized to synthesize nanoparticles. Initially, we dissolved 10 wt% of ZnCl_2_ in DMF at room temperature while stirring. Following this, we added PVP, glucose, NaOH, and PES to the solution according to the specifications outlined in Table 1. The mixtures were then heated in a Teflon cell in the microwave for 20 min at 80 °C before being cooled down to room temperature. To separate the suspensions into two phases, we centrifuged them at 6000 rpm for 15 min and transferred the contents into falcon tubes.

The solid phase collected after centrifugation was washed with ethanol and water, followed by drying in an oven at 65 °C.

Table 1 summarizes the compositions used to prepare the different nanoparticle solutions.

### 2.3. Membrane Preparation

The porous polymeric PES-ZnO nanocomposite membranes were fabricated using the phase inversion method, as outlined in reference [27]. Table 2 displays the percentages of the different mixtures utilized in the manufacturing process. The casting solution contained mainly a 15% wt PES powder dissolved in DMF. All mixtures except for PES-5 underwent 20 min of microwave treatment. After preparing the homogeneous solution, it was rested for one hour to degas before casting. The solution was cast onto a glass plate and evenly spread using a 200 µm thick doctor’s blade. Subsequently, the glass plate was submerged in a coagulation bath filled with distilled water for 1 h.

Subsequently, the membranes were annealed twice, each time for 10 min in a distilled water bath at 60 °C. This thermal treatment was carried out to eliminate the solvent and the additive.

### 2.4. Nanoparticle Characterization

The synthesis of ZnO-NPs was monitored using a UV–Visible spectrometer UV-1900 Shimadzu (Shimadzu, Kyoto, Japan), with UV–Vis analysis conducted on powders dispersed in water. The particle sizes of the synthesized ZnO-NPs were measured using Dynamic Light Scattering (DLS), Zetasizer Malvern NanoZS (Malvern Panalytical Ltd., Malvern WR14 1XZ, UK).

The crystallite size was estimated from the XRD diffractogram using the Scherrer equation, which is presented as Equation (1).

(1) $$<D>=\frac{k\lambda_{Cu}}{\beta_{corr}\times cos\theta }$$

where <D> represents the average crystallite size β_corr_ = β_exp_ − β_ref_; β_exp_ is the broadened profile width of the experimental sample; β_ref_ is the standard profile width of the reference silica sample; k is the shape factor (k = 0.94); and 2θ corresponds to the positions of the eight most intense peaks.

### 2.5. Membrane Characterization

The surface morphology of the membrane was examined using the TESCAN VEGA SEM system (Tescan, Brno, Czechia). Before observation, specimens were sputter-coated with gold.

In this study, we used the AFM model CoreAFM from Nanosurf (Gräubernstrasse, Liestal, Switzerland) to determine the surface morphology (2D and 3D topographic images). We used an aluminum cantilever to scan the membranes and employed the phase imaging mode at room temperature in an air medium to characterize the membranes. When imaging the membrane structure, we captured the samples without any preparative procedures by simply attaching them to a steel disc with double-sided tape. We measured the mean roughness (Ra) and the root mean square of data (Rq) of the developed membranes in a scan area of 10 µm × 10 µm.

To measure the membrane’s hydrophilicity, we used an Attension Theta blood pressure monitor T200 with an optical camera. We managed the monitoring process using the “One Attension Software, Version 4.03.(r8310)”. We recorded contact-angle measurements and calculated the corresponding standard deviation to obtain the mean value.

The porosity (Ɛ %) was estimated via the gravimetric method. The porosity is defined as the ratio of the voids’ volume in the membrane (V_v_) to the volume of the membrane plus voids (V_v_ + V_m_). Before impregnating the dry membrane in kerosene, for 24 h, its mass (m_d_) was measured. After removing the excess kerosene, the mass (m_w_) of the wet membrane was determined. The porosity (Ɛ) was calculated using Equitation (2):

(2) $$Ɛ\left(\%\right)=\frac{V_{v}}{V_{v}+V_{m}}\times 100=\left\{\frac{(m_{w}-m_{d})/\rho_{k}}{(m_{w}-m_{d})/\rho_{k}+\frac{m_{d}}{\rho_{p}}}\right\}\times 100$$

where m_w_ is the mass of the wet membrane, m_d_ is the mass of the dry membrane, ρ_p_ is the density of the polymer (1.28 g·cm^−3^) and ρ_K_ is the density of kerosene (0.82 g cm^−3^).

The water uptake (WU) of the synthesized membrane was determined according to Equation (3):

(3) $$WU=\frac{m_{wm}-m_{dm}}{m_{wm}}\times 100$$

where m_wm_ is the mass of the wet membrane obtained after the excess water on the membrane surface was wiped. The mass of the dry membrane (m_dm_) was measured after drying the membrane, in a ventilated oven at 70 °C overnight.

The membrane’s permeability was assessed using a stainless-steel cell (CM Celfa P28) with an effective membrane area equal to 28.26 cm^2^.

The membrane permeate flux (J_v_) was measured as a function of transmembrane pressure ranging from 0 to 12 bar. The flux, J_v_ (L·m^−2^·h^−1^), was determined using the following equation:

(4) $$J_{v}=\frac{V}{A\cdot ∆t}$$

where A (m^2^) is the membrane’s effective area, V (L) is the permeate volume, and ∆t (h) is the filtration time.

Moreover, it is possible to calculate the retention via the Equation (5):

(5) $$R\left(\%\right)=\left(1-\frac{C_{p}}{C_{f}}\right)\times 100$$

where C_f_ and C_p_ are the feed and the permeate dye concentrations, respectively.

### 2.6. Antifouling Property

To evaluate the effectiveness of the membranes’ ability to resist fouling, a solution containing bovine serum albumin (BSA) at a concentration of 100 mg/L was utilized. The decline in flux ratio was continuously monitored during the filtration of the BSA solution, which enabled a thorough analysis of the membrane’s antifouling behavior. To accurately assess the effectiveness of the membrane’s antifouling properties, we calculated various parameters.

(6) $$FRR\left(\%\right)=\frac{J_{v2}}{Jv}\times 100$$

(7) $$Rr\left(\%\right)=\frac{{(J_{v2}-J}_{vB})}{Jw}\times 100$$

(8) $$Rir\left(\%\right)=\frac{{(J_{v}-J}_{v2})}{Jwv}\times 100$$

(9) $$Rt\left(\%\right)=Rr+Rirr=\left(1-\frac{{Jv}_{2}}{J_{v}}\right)\times 100$$

where J_v2_ represents the recovered flux, while J_vB_ indicates the flux of BSA solution. FRR (%) stands for the flux recovery ratio, which measures the effectiveness of the antifouling feature after one cycle. R_r_ (%) represents the reversible flux decline ratio caused by fouling, which can be eliminated after membrane cleaning with water. R_ir_ (%) estimates the irreversible flux decline ratio, and R_t_ (%) indicates the flux decline ratio caused by BSA fouling [11].

### 2.7. ZnO Nanoparticle Leakage During NIPS

In order to analyze the leakage of ZnO-NPs during the NIPS process, we implemented a straightforward technique. This involved adding 9 g of water to 1 g of the nanocomposite polymeric solution, leading to the coagulation of the polymeric solution and the subsequent dissolution of the solvent (DMF) into the water. Following this, we employed atomic absorption spectrometry to accurately measure the presence of Zn in the resulting water–DMF solution. This analytical approach enabled us to precisely determine the quantity of leaked Zn, facilitating accurate calculations of the Zn content retained by the membrane.

### 2.8. Antibacterial Activity

In this study, *Staphylococcus aureus* (Gram-positive, ATCC 25923) and *Escherichia coli* (Gram-negative, ATCC 25922) were used to evaluate the antibacterial activity. Nutrient Agar medium (Sigma-Aldrich) was prepared according to the manufacturer’s instructions and poured into disposable, sterilized Petri dishes to provide a consistent growth substrate. After solidification, each Petri dish was inoculated with the respective bacterial strain under aseptic conditions. The plates were then incubated in an incubator at 37 °C for 24 h to promote optimal bacterial growth. This setup allowed for a controlled environment to assess bacterial responses on the prepared medium.

## 3. Results and Discussions

### 3.1. Nanoparticle Characterization

**Figure 1:**
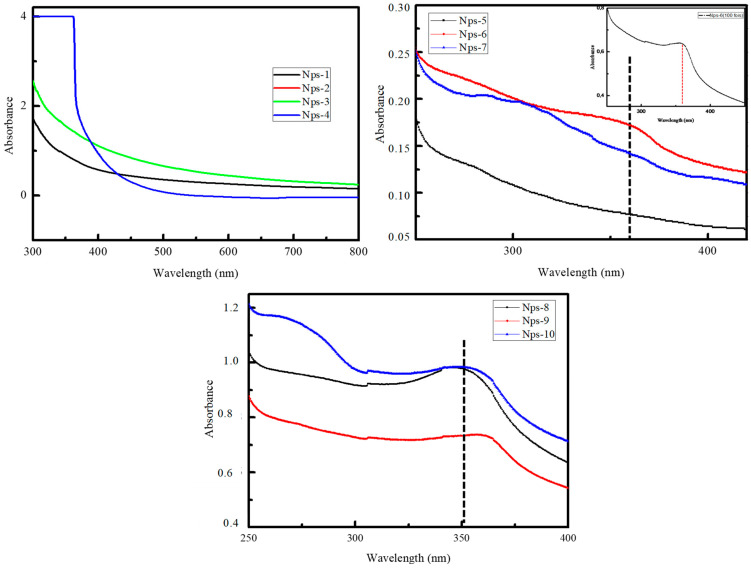
UV–Vis spectrum of ZnO prepared via microwave method.

ZnO-NPs exhibit unique optical properties that UV–Vis spectroscopy can effectively study. This technique allows for examining the optical absorption characteristics of ZnO-NPs, which provides information about their size, shape, and distribution. Previous studies found that ZnO-Nps have a characteristic absorption peak in the UV–Visible range, which was recorded between 355 to 385 nm, as shown in Table 3. Figure 1 shows the UV–Visible spectra of ZnO-NPs dispersed in water. In Figure 1, it is evident that there were no peaks related to the ZnO nanoparticle when NaOH was not added (NPs-1 to NPs-4). However, after the addition of NaOH (NPs-6), a peak around 360 nm appeared, indicating the successful synthesis of ZnO-NP. The addition of NaOH is essential for the synthesis of ZnO-NPs, as verified by UV–Visible analysis.

This important step should not be overlooked to ensure successful nanoparticle synthesis. The microwaves provide rapid, uniform heating, which facilitates the in situ conversion of ZnCl_2_ to ZnO-NPs through the following reaction:

$$ZnCl_{2}\overset{Microwave}{\to }ZnO+2HCl$$

Upon the introduction of glucose (NPs-7), smaller ZnO-NPs are produced, as indicated by the blue shift towards the shorter wavelength (approximately 320 nm). Figure 1 demonstrates that the addition of PES (NPs-8 and NPs-10) results in a blue shift (approximately 350 nm) and an increase in the intensity of the absorption peak. The blue shift suggests that the ZnO-NPs may be interacting with the sulfonyl groups of PES, forming a strong interface. Moreover, the increased intensity of the absorption peak indicates that the dispersion of ZnO-NPs is uniform within the PES matrix [28,29,30].

**Table 3:** The UV–Vis absorption peaks of ZnO-NPs from the literature.

The Authors	ƛ_max_ (nm)	Reference
N. M. Shamhari et al.	357	[31]
M. Pudukudy et al.	378	[32]
P. Fageria et al.	369	[33]
S. Fakhari et al. (bulk)	385	[28]
S. Fakhari et al. (NPs)	350	[28]
A. Samy et al.	376	[34]
C. Taranath et al.	374	[35]
A. M. Ismail et al.	370	[36]
B. Bulcha et al.	376	[37]
In this work	356	-

**Figure 2:**
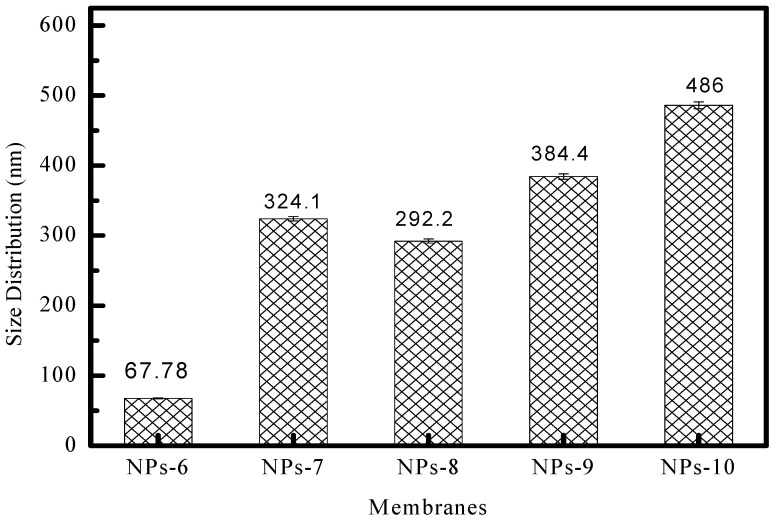
The sizes of the nanoparticles obtained by DLS.

The ZnO-NPs that we synthesized were carefully analyzed using Dynamic Light Scattering (DLS), and the detailed results are illustrated in Figure 2. This figure vividly demonstrates that the size of the ZnO-NPs measures approximately 68 nm when PES is not included in the process. However, when PES is introduced, there is a noticeable increase in particle size, attributed to the encapsulation of ZnO-NPs by the PES polymer. These findings are further corroborated by the results obtained from the UV–Visible technique.

**Figure 3:**
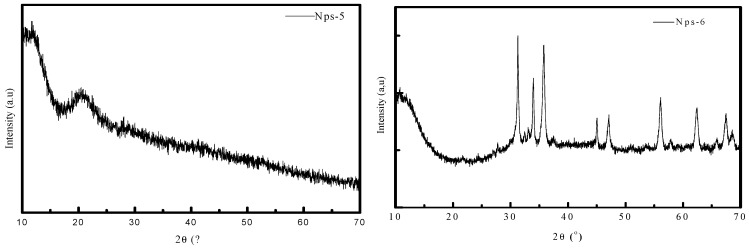
XRD of the ZnO-NPs. The XRD analysis illustrates the structural differences between ZnO-NPs before (Nps-5) and after (Nps-6) the addition of NaOH. The left side (Nps-5) exhibits no characteristic ZnO peaks, indicating an absence of crystalline ZnO formation. In contrast, the right side (Nps-6) shows well-defined ZnO diffraction peaks, confirming successful nanoparticle synthesis facilitated by NaOH addition.

The crystallinity of the synthesized NP specimens was examined via XRD. The diffractograms related to NPs-5 and NPs-6 are shown in Figure 3 respectively. The XRD diffractograms of NPs-5 (without NaOH) and NPs-6 (with NaOH) indicate that ZnO-NPs are only formed after the addition of NaOH. This result is consistent with the findings from UV–Visible and Dynamic Light Scattering. The identified XRD peaks at 31.35°, 34.04°, 35.87°, 47.09°, 56.12°, 62.49°, 68.1° and 67.50° correspond to the (1 0 0), (0 0 2), (1 0 1), (1 0 2), (1 1 0), (1 0 3), (1 1 2), and (2 0 1) reflections, respectively. These strong and unambiguous diffraction peaks show that the ZnO-NPs have good crystallinity and are coordinated with the hexagonal phase, compared to the literature. Identical results for ZnO-NPs have also been confirmed in the literature [30,38]. The product exhibits a fine crystalline structure, evidenced by sharper and stronger diffraction peaks. The average crystallite size of ZnO-NPs was estimated to be 40.7 nm using Scherrer’s formula.

### 3.2. Membrane Characterization

**Figure 4:**
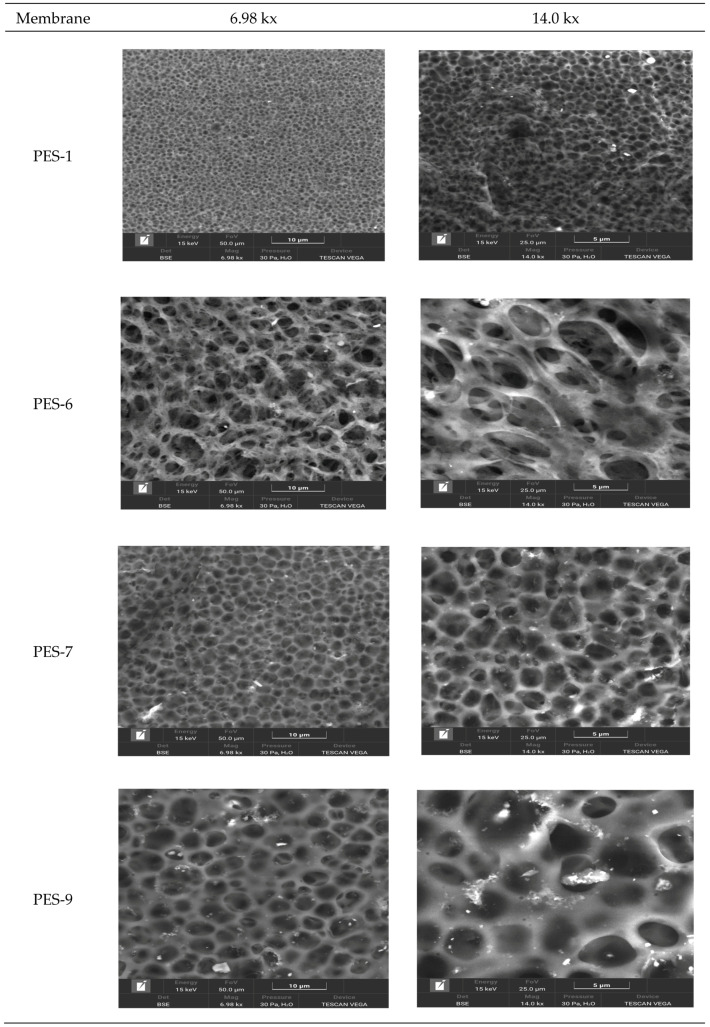
SEM images of the membranes.

Figure 4 displays SEM images of various membranes: PES-1 (raw PES), PES-6 (1% ZnCl_2_ without NaOH), PES-7 (1% ZnCl_2_ with NaOH), and PES-9 (5% ZnCl2 with NaOH), shown at two different magnifications. These SEM images offer important insights into the morphology, distribution, and surface characteristics of the PES-ZnO (polyethersulfone–zinc-oxide) nanocomposite membranes. The images show that the inclusion of ZnO-NPs results in a more heterogeneous surface. Generally, ZnO-NPs are dispersed throughout the PES matrix, which enhances the membrane’s roughness and may influence its surface wettability and antifouling behavior. The SEM images of PES-7 and PES-9 demonstrate a uniform dispersion of ZnO-NPs. However, the PES-9 image reveals small distinct clusters, or “islands”, indicating the uneven distribution and aggregation of ZnO. These clusters could affect the membrane’s mechanical strength, permeability, and rejection efficiency. In contrast, the PES-6 image displays an increase in the membrane’s surface pore size, which is unevenly distributed.

Adding NaOH in the dope solution for PES-7 and PES-9 leads to the formation of ZnO-NPs within the membrane matrix, resulting in a uniform dispersion of the membrane’s surface pore size. Increased ZnO content leads to larger pores, impacting the balance between selectivity and permeability in filtration applications.

The SEM images also indicate that the membrane surface becomes more textured after the addition of ZnO-NPs, contributing to increased surface roughness. This increased roughness can enhance hydrophilicity, as ZnO is generally hydrophilic, which subsequently improves water affinity and potential antifouling properties. However, excessive roughness might lead to higher particle deposition on the surface during filtration, potentially counteracting some antifouling benefits.

**Figure 5:**
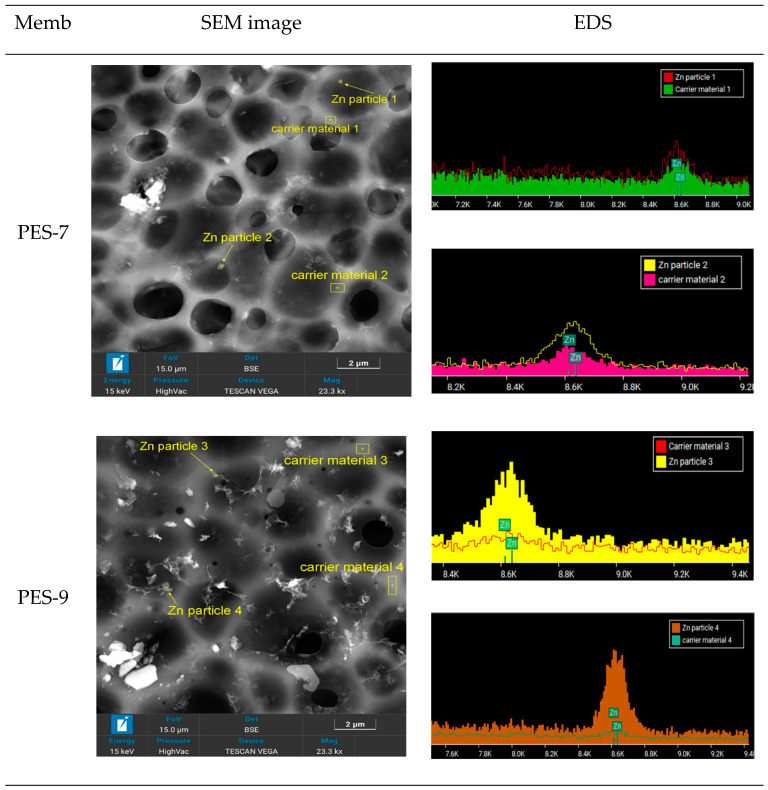
EDS analyses of PES-ZnO nanocomposite membranes.

The EDS results for the PES-ZnO nanocomposite membranes, synthesized using microwave-assisted techniques, are illustrated in Figure 5. The detection of Zn peaks—present in a homogeneous and expected quantity—confirms the formation of ZnO-NPs within the PES structure during the microwave synthesis process. The EDS analysis verifies the incorporation of Zn into the PES matrix, indicating the successful integration of ZnO. The SEM images demonstrate a uniform distribution of Zn throughout the membrane, which ensures consistent membrane performance. If the Zn were distributed non-uniformly or in clusters, it could suggest the aggregation of ZnO particles, potentially resulting from either an uneven synthesis process or excessive ZnO loading. Proper dispersion is crucial for maximizing the surface area of ZnO exposure, thereby enhancing properties such as hydrophilicity and antimicrobial activity.

As confirmed by both the SEM and EDS analyses, the successful in-situ incorporation and homogeneous dispersion of ZnO within the PES matrix are essential for achieving the desired improvements in the membrane’s properties. A uniformly distributed ZnO contributes to consistent antimicrobial effects, increased hydrophilicity, and potentially improved fouling resistance. Furthermore, the presence of ZnO without excessive clustering helps maintain the membrane’s structural integrity and permeability [39].

**Figure 6:**
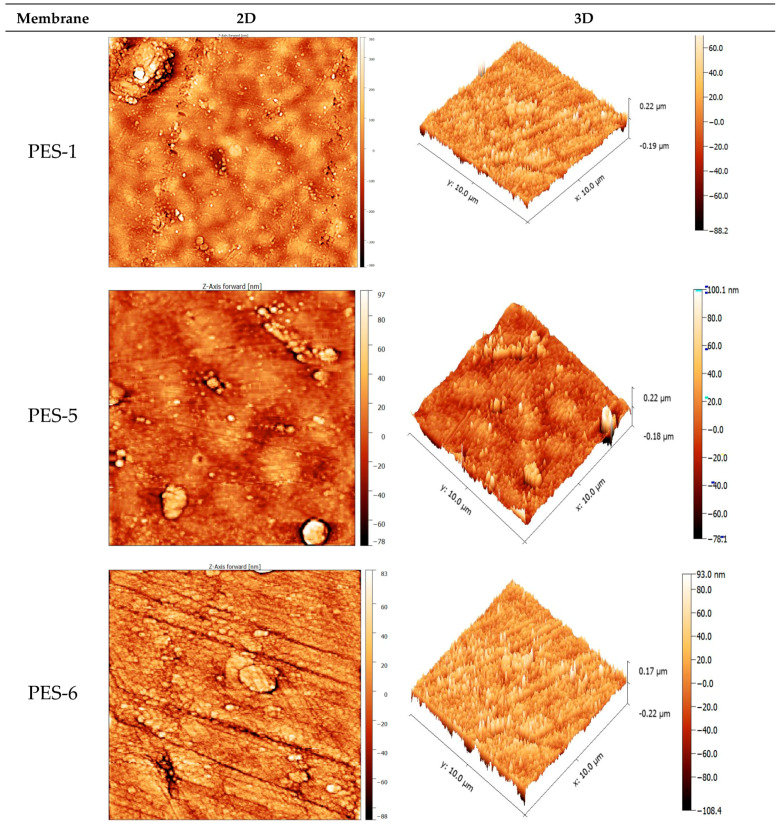

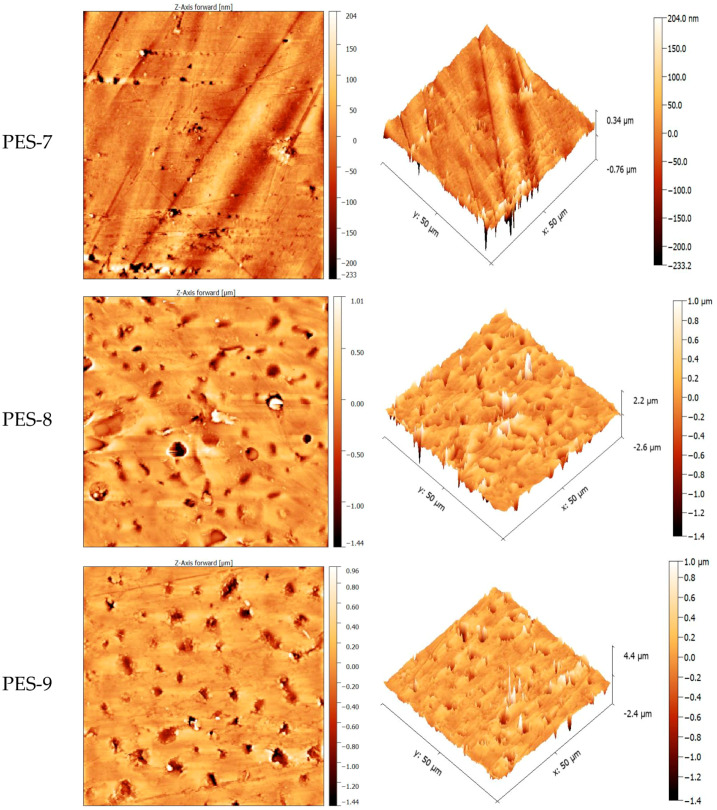
AFM images of the PES-ZnO nanocomposite membranes.

Furthermore, AFM was used to illustrate the surface roughness of PES-ZnO membranes synthesized by microwave-assisted process. The results are presented in Table 4 and Figure 6. For PES-1, the surface roughness was 108.6 nm, which probably resulted from film shrinkage during the NIPS process. Apart from the elevated roughness, PES-1, PES-5, and PES-6 (in the absence of ZnO-NPs) exhibit lower surface roughness than PES-7, PES-8, and PES-9 in which the ZnO-NPs are present. After incorporating ZnO-NPs, membrane surface roughness increased from 52.9 nm for PES-7 to 129.2 nm for PES-9. This confirms that after microwave treatment, the formation of nanoparticles leads to a rough membrane surface on PES. Various studies have reported that the incorporation of nanoparticles increases membrane surface roughness [40,41]. Wenzel’s equation [39] suggests that surface hydrophilicity/hydrophobicity can be enhanced by roughness. Generally, for hydrophilic membranes with identical chemical textures, the contact angle would decrease if the surface were rough. The addition of nanoparticles was observed to influence surface morphology and hydrophilicity, as evidenced by water contact-angle measurements. Excessive nanoparticle addition resulted in aggregation, consistent with SEM observations.

**Figure 7:**
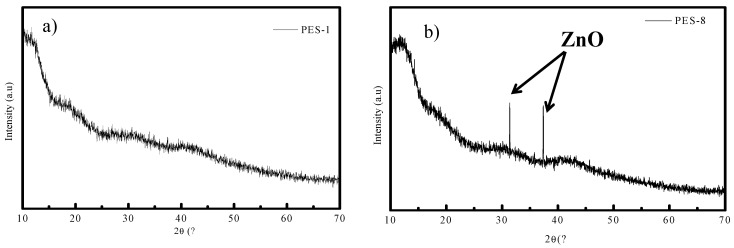
XRD diffractograms of (**a**) the neat membranes and (**b**) the PES-ZnO nanohybrid membrane.

XRD analysis was performed in this study to approve the presence of embedded nanomaterial in the polymeric membrane. Figure 7a,b display the XRD diffraction patterns of the neat PES membrane (PES-1) and PES-ZnO nanocomposite membrane (PES-8), respectively. Indeed, in Figure 7a, no peak was recorded, which clearly shows the amorphous nature of the PES structure. However, in Figure 7b, two small and sharp peaks were recorded. These two peaks, which appear at 31.77° and 36.25°, were assigned to the ZnO-NPs, embedded in the PES membrane. There was a discernible shift in the maximum peak between the ZnO nanoparticle diffraction patterns in the PES polymer and pure ZnO, indicating an interaction between the ZnO-NPs and the PES polymer. The results demonstrate that the ZnO-NPs were successfully embedded into the PES polymer to create a nanohybrid membrane. Moreover, the results confirm that ZnO has a strong affinity with PES polymer, thereby preventing the leaching of nanoparticles during the phase inversion process [42].

**Figure 8:**
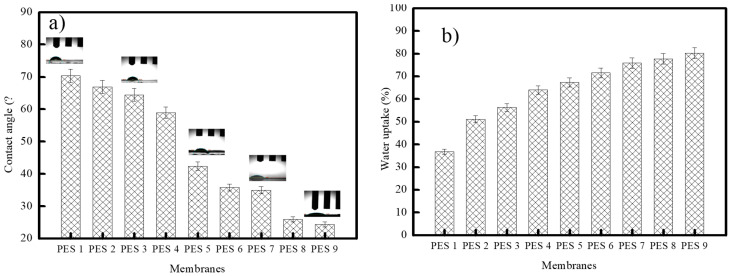
(**a**) The contact angle; and (**b**) water absorption of the fabricated membranes.

Improving the wettability by increasing the membrane’s hydrophilicity can increase the water flux and may influence the permeability and the antifouling characteristics. The contact angle and water uptake are presented in Figure 8. As the results show, the contact angle decreased from 70.33 to 24.4 for the membrane PES-1 to PES-9. The observed decrease in contact angle during the in-situ synthesis of PES-ZnO nanocomposite membranes, especially when microwaves are used, can be attributed to several factors related to the surface characteristics and modification processes of the nanocomposite. Incorporating ZnO-NPs into the PES matrix alters the surface energy of the membrane. ZnO typically has a high surface energy [43], which enhances the hydrophilicity of the composite when integrated into the hydrophobic PES matrix, resulting in a reduced contact angle. Additionally, the application of microwave radiation during synthesis promotes a more uniform distribution and better dispersion of ZnO-NPs within the PES matrix. This improved dispersion increases interactions between the hydrophilic ZnO and the membrane surface, facilitating water uptake and further lowering the contact angle. It is also noteworthy that incorporating ZnO-NPs can increase the surface roughness of the membranes. This increased roughness can lead to the Cassie–Baxter state, where air pockets form on structured surfaces. This phenomenon affects water droplet behavior and contributes to a decrease in the effective contact angle. Furthermore, interactions between the hydroxyl groups on the ZnO surface and the functional groups of PES enhance wettability, creating a more favorable surface for water droplet spreading. Overall, the hydrophilic properties of ZnO-NPs can significantly improve the hydrophilicity of the PES-ZnO membranes during synthesis or modification. This decrease was attributed to the formation of the hydrophilic sites COOH and SO_3_H on the surface of the polymer. Since ZnO is hydrophilic and has a higher affinity for water [44,45], the decrease in contact angle can also be due to incorporating ZnO-NPs in the membrane. Moreover, ZnO can enhance the membrane’s accessibility for additional hydrogen bond interactions, enhancing water flow across them [46].

The water absorption variation has a similar trend to the contact angle. The maximum water absorption recorded was 77% for the membrane with the highest ZnO-NP content; however, the minimum water absorption, 36%, was recorded for the unmodified membrane.

**Figure 9:**
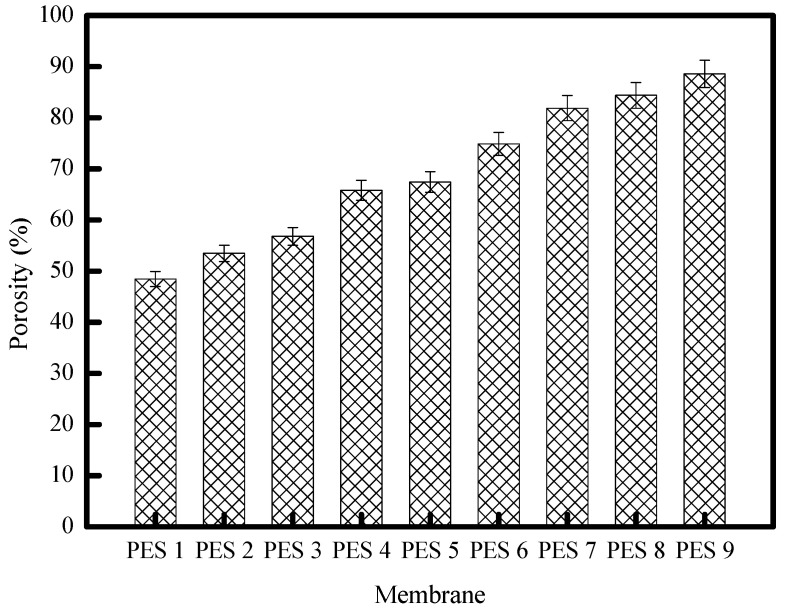
The porosity of the PES-ZnO membranes.

The results of the gravimetric measurements of the membrane’s porosities are illustrated by the histograms in Figure 9. These results show that the porosity is proportional to the amount of ZnO incorporated in the pure PES membrane. The high ZnO affinity for water can explain this increase in porosity. Thus, it can easily suck water into the casting slurry and promotes the formation of macro-voids, which contribute to the porosity improvement [13].

**Figure 10:**
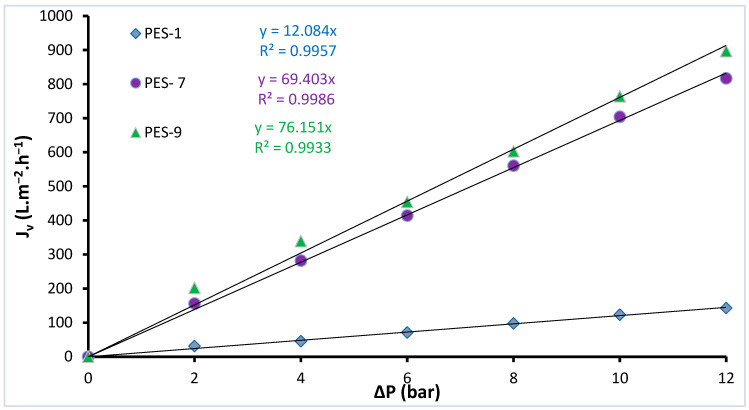
The effect of pressure on pure water flux for the PES-ZnO membranes.

We thoroughly examined the impact of ZnO-NPs on the permeability of the fabricated membranes, applying Darcy’s law (Jv = Δp. Lp) to assess flux variations with pressure. The results, illustrated in Figure 10 and Table 5, demonstrate a clear trend: as the ZnO-NP concentration increased, so did the membrane permeability. This enhancement can be interpreted through the synergistic influence of two key factors—hydrophilicity and porosity. ZnO-NPs are known to introduce polar functional groups within the polymer matrix, thereby increasing hydrophilicity. This property enhances water affinity, allowing water molecules to interact more effectively with the membrane surface, reducing resistance to flow. Additionally, the inclusion of ZnO-NPs modifies the structural arrangement of the polymer matrix, enlarging the pore size and enhancing interconnectivity, thereby improving water passage through the membrane.

An interesting observation was the superior performance of membrane PES-6 compared to membrane PES-5, despite their nearly identical compositions. This discrepancy can be attributed to the microwave treatment applied to the PES-6 dope solution before the NIPS process. The microwave exposure likely generated free radicals, which acted as reactive sites, enabling the more effective integration of ZnO-NPs into the polymer network. This improved integration not only enhanced nanoparticle dispersion but also led to a more uniform distribution of pores and a robust network, further boosting membrane permeability. The findings underscore the dual role of ZnO-NPs in improving both the hydrophilic properties and the porosity of the membranes, which together contribute to enhanced water flux. Additionally, the study highlights the importance of pre-treatment methods, such as microwave exposure, in optimizing nanoparticle incorporation and tailoring membrane properties for specialized applications. This multi-faceted approach provides valuable insights for the development of high-performance membranes designed for demanding filtration and separation processes.

It is important to note that the flux values observed in this study are lower than those reported in our previous work [13], where the PVP concentrations were significantly higher (10%, 15%, and 20%) compared to the 1% PVP used here. PVP is well-known for its pore-forming ability, and higher concentrations lead to increased porosity and hydrophilicity, resulting in greater flux. The reduced PVP concentration in this study was intentionally chosen to investigate the isolated effects of ZnO nanoparticles (ZnO-NPs), offering valuable insights into their role in membrane performance. These findings highlight the dual role of ZnO-NPs in enhancing both hydrophilic properties and porosity, which in turn improves water flux. Additionally, the study emphasizes the importance of pre-treatment methods, such as microwave exposure, for optimizing nanoparticle integration and tailoring membrane properties for specific applications. By comparing the current results to our previous work, this study provides a deeper understanding of the interaction between pore-forming agents and nanoparticles in developing high-performance membranes for advanced filtration and separation processes.

**Figure 11:**
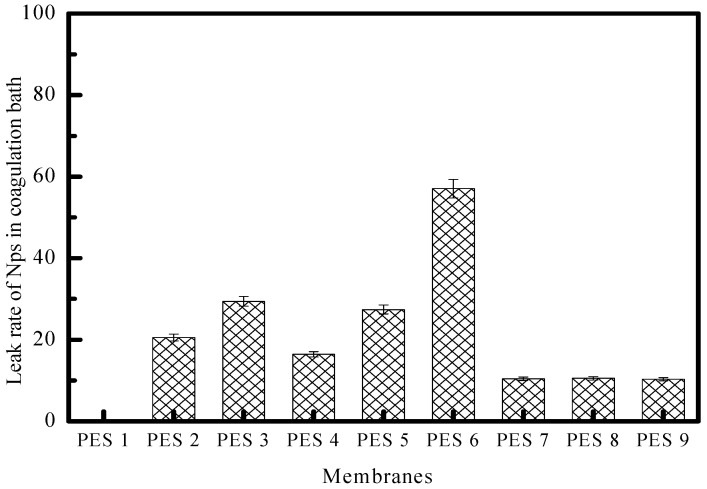
Percentages of ZnO’s leakage from the prepared composites.

Figure 11 underscores the critical relationship between ZnO nanoparticle leaching and the stability of PES-ZnO composites, where lower percentages of leached ZnO reflect improved integration within the polymer matrix. The data in Figure 11 suggests that the most stable composites are achieved from alkaline mixtures treated with microwave-assisted synthesis, stabilizing the ZnO by modifying its surface and reducing oxygen vacancies. Microwave-assisted synthesis emerges as a key technique in enhancing composite stability, as it not only improves nanoparticle dispersion but also reduces surface defects like oxygen vacancies on ZnO particles, thereby strengthening their compatibility with the PES matrix. These modifications are critical because weak particle–matrix interactions, uneven ZnO distribution, or excessive porosity during the phase inversion process can exacerbate nanoparticle leakage into the coagulation bath. While phase inversion solidifies the PES matrix, it also represents a point of vulnerability, as the kinetics of polymer precipitation, the nanoparticle–polymer affinity, and the porosity of the resultant membrane influence leaching.

Microwave-assisted synthesis mitigates these challenges by enhancing nanoparticle integration, but further improvements can be achieved through strategies such as the surface functionalization of ZnO to increase chemical bonding with PES, controlling the phase inversion rate to balance solidification and particle encapsulation, and incorporating crosslinking agents to reinforce structural stability. Such measures not only minimize environmental risks associated with nanoparticle leaching, but also preserve the composite’s critical antifouling, antibacterial, and self-cleaning properties, which are essential for applications like water treatment. By refining these processes, PES-ZnO composites can achieve greater durability, optimized performance, and sustainability, demonstrating the potential for advanced material engineering to address both functional and ecological considerations.

**Figure 12:**
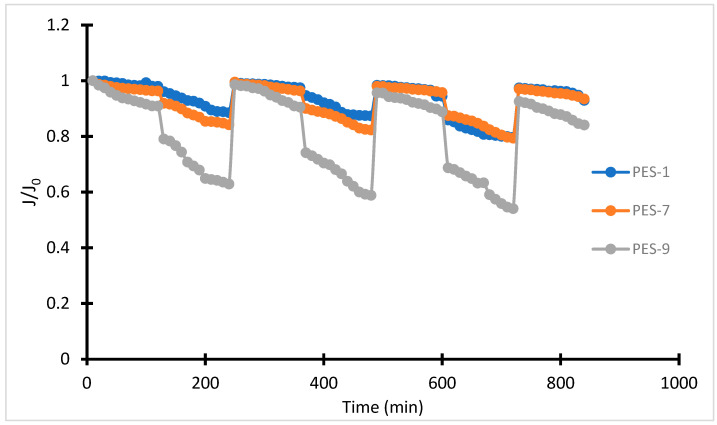
Water fluxes as a function of time throughout three cycles of the BSA UF testing of the prepared PES-ZnO membranes.

Evaluating flux decay is pivotal for understanding the antifouling properties of membranes, particularly when exposed to challenging feed solutions. Figure 12 reveals that increasing the content of ZnO-NPs within PES membranes enhances water flux. This enhancement arises from the positive impact of ZnO-NPs on the membrane’s hydrophilicity and porosity, which together improve water permeability. However, introducing bovine serum albumin (BSA) into the feed solution reduces water flow due to fouling mechanisms like concentration polarization and pore blockage. These phenomena lead to a “sealing” effect, wherein protein accumulation forms a barrier, decreasing flux and necessitating antifouling strategies.

**Figure 13:**
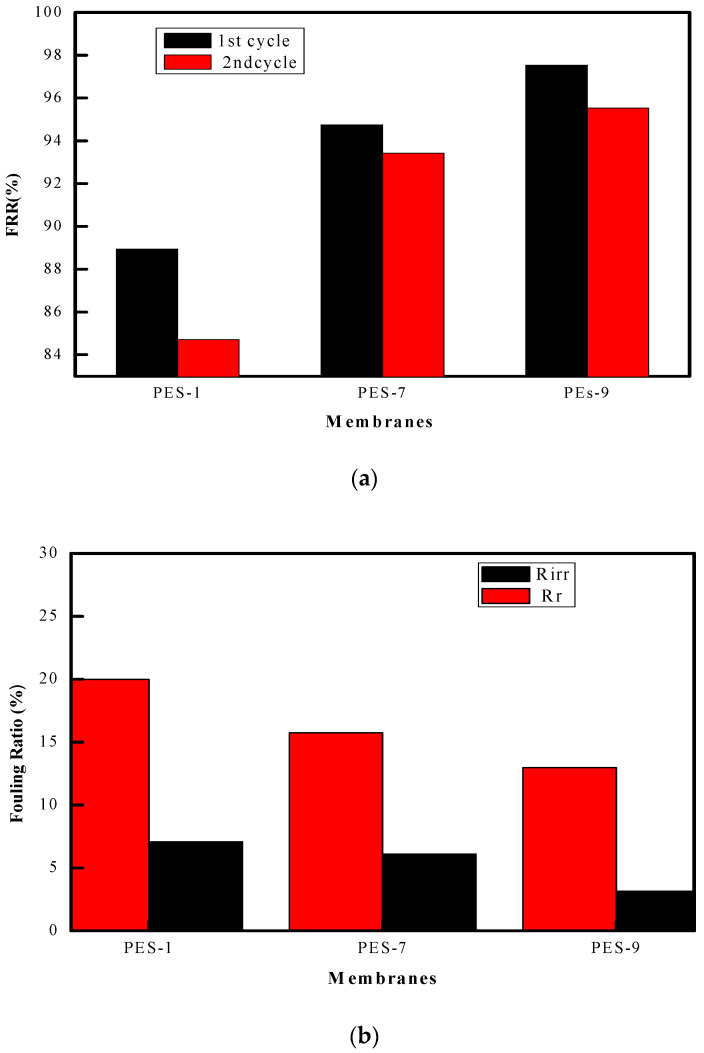
(**a**) The FRR percentage results of the membranes throughout the first two cycles. (**b**) Percentage of the total fouling ratio (Rt); and the irreversible fouling ratio (Rirr).

Further insights into fouling behavior are illustrated in Figure 13, which reports reversible fouling rates (Rr), irreversible fouling rates (Rir), and total fouling (Rt). An increase in ZnO-NP content correlates with a higher flux recovery rate (FRR), indicating improved antifouling efficiency. For instance, during the first ultrafiltration cycle, FRR increased from 88.93% in PES-1 to 97.52% in PES-9, demonstrating that ZnO incorporation significantly facilitates recovery [11]. The second cycle exhibited a similar trend, underscoring the durability of the antifouling improvements.

Interestingly, total fouling (Rt) decreased from 19.98% in pure PES membranes to 12.96% in ZnO-enriched PES-9 membranes. This reduction was primarily attributed to a decrease in reversible fouling (Rr), likely due to the accumulation of BSA forming a superficial cake layer. This layer is less adhesive on ZnO-integrated membranes, allowing for easier hydraulic cleaning and flux recovery. Irreversible fouling (Rir), while also reduced, remains a challenge, emphasizing the need for further advancements in nanoparticle integration and surface engineering for long-term applications [13].

Including ZnO-NPs in PES membranes fundamentally alters their surface characteristics, particularly by enhancing hydrophilicity. Hydrophilic surfaces are less prone to the adsorption of hydrophobic contaminants like BSA, mitigating initial fouling. Moreover, ZnO’s photocatalytic properties enable the generation of reactive oxygen species (ROS) under suitable conditions, facilitating the breakdown of foulants and promoting self-cleaning. This dual mechanism—physical resistance to fouling and chemical self-cleaning—substantially enhances the membrane’s antifouling capabilities.

While the study highlights significant progress in mitigating reversible fouling and achieving efficient hydraulic cleaning, irreversible fouling remains a limiting factor for long-term operation. To address this, future research could explore advanced crosslinking techniques, surface modifications, or hybrid membrane systems to optimize the integration of ZnO-NPs and further enhance membrane performance. These insights collectively demonstrate the potential of ZnO-enriched membranes for sustainable and efficient water treatment applications.

**Figure 14:**
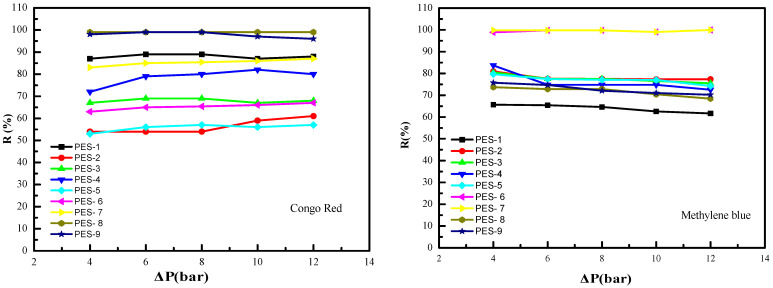
Effect of pressure on dye retention. Left side (Congo Red), Right side (Methylene Blue).

The performance of polyethersulfone (PES)-ZnO hybrid membranes in dye retention was systematically evaluated using two representative dyes, methylene blue (MB) and Congo red (CR). Figure 14 illustrates the retention rates (R%) of these dyes as a function of applied pressure (ΔP), with the results showing a significant improvement in retention as pressure exceeded 4 bar. This suggests that a higher driving force enhances the filtration efficiency, likely by promoting the tighter packing of dye molecules on the membrane surface and improved rejection through size-exclusion and adsorption mechanisms.

The incorporation of ZnO-NPs into the PES matrix substantially enhanced dye retention rates, with increases of 15.3% for MB and 12.6% for CR. This improvement can be attributed to several synergistic effects introduced by ZnO. First, ZnO-NPs possess a high surface area and intrinsic adsorption capacity, enabling them to effectively adsorb dye molecules such as MB and CR. The literature supports ZnO’s affinity for these dyes, as the nanoparticles interact through electrostatic attractions and hydrogen bonding, contributing significantly to dye retention [5,46,47,48].

Additionally, ZnO incorporation was observed to enhance both the porosity and hydrophilicity of the PES-ZnO membranes, as confirmed by contact-angle measurements [49]. Increased porosity improves the structural pathways for water and solute transport, facilitating higher retention by intercepting dye molecules. Enhanced hydrophilicity reduces the fouling potential of hydrophobic dyes like MB and CR by decreasing their adhesion to the membrane surface, thereby promoting their rejection.

Furthermore, the membrane’s selective barrier properties may be influenced by the uniform distribution of ZnO within the PES matrix, which optimizes pore size and surface charge characteristics. This uniformity not only supports size-exclusion mechanisms but also creates an electrostatic barrier against negatively charged CR molecules or positively charged MB molecules under certain operating conditions.

These findings underscore the importance of ZnO as a functional additive in PES membranes, demonstrating its dual role in improving both adsorption-driven and filtration-driven dye retention mechanisms. The results highlight the potential for PES-ZnO hybrid membranes in applications requiring the removal of dyes and organic pollutants, particularly in wastewater treatment, where membrane performance and longevity are critical. Future studies could explore the interplay between ZnO loading, dye-specific interactions, and long-term operational stability to further optimize these membranes for industrial applications.

**Figure 15:**
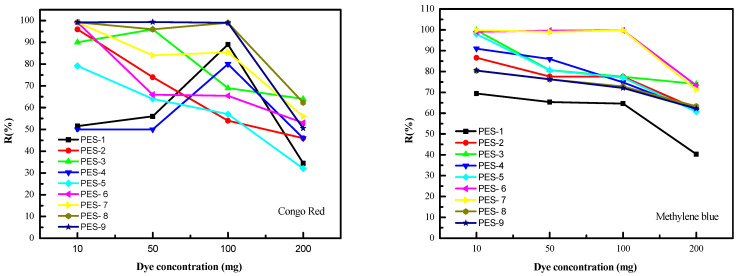
Effect of initial dye concentration. Left side (Congo Red), Right side (Methylene Blue).

The initial dye concentration in the feed solution was studied, since it may have an important effect on the retention rate. The variation of the retention rate with the initial dye concentration is shown in Figure 15. As observed in Figure 15, the retention rate for all the prepared PES-ZnO hybrid membranes initially increases to a maximum before declining. The decrease in retention rate can be attributed to the rise in osmotic pressure as the dye concentration increases, which causes concentration polarization and results in the formation of a thin dye layer on the membrane surface.

**Figure 16:**
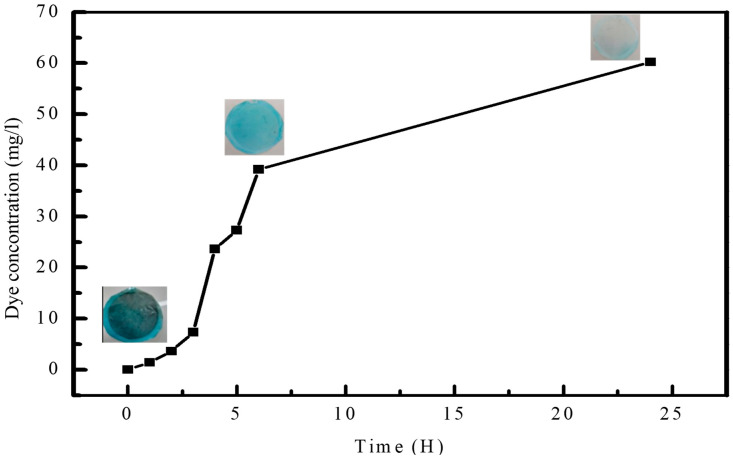
Self-cleaning of the membrane.

To evaluate the effectiveness of the membrane’s self-cleaning properties, it was subjected to sunlight. Figure 16 depicts the outcome of the photocatalytic degradation of MB by the membranes after being exposed to sunlight. The results indicate that ZnO-NPs are capable of quickly breaking down MB under solar radiation. The PES-9 membrane, which has the highest concentration of ZnO, exhibited the greatest degree of degradation under UV light. During the irradiation process, the photodegradation of MB occurred gradually. According to studies [47,48], it seems that the nitrogen and sulfur atoms of MB molecules were converted into ions and gases during irradiation exposure. The integration of (ZnO-NPs) into the PES matrix through microwave-assisted synthesis allows the membranes to leverage the photocatalytic properties of ZnO, which is essential for effective self-cleaning. ZnO is a well-known photocatalyst with a wide band gap (approximately 3.37 eV), which enables it to generate ROS such as superoxide anions (·O^2−^) and hydroxyl radicals (·OH) under UV irradiation. When exposed to UV light, ZnO-NPs in the PES matrix absorb photons and generate electron–hole pairs. These electrons and holes migrate to the surface, where they interact with oxygen and water molecules to form ROS. These reactive species possess strong oxidative abilities, capable of degrading organic contaminants, including dyes, oils, and bioorganic foulants that typically accumulate on membrane surfaces.

The use of microwave-assisted synthesis for the in-situ incorporation of ZnO-NPs in the PES matrix ensures uniform dispersion and strong adhesion of ZnO to the polymer structure. This uniform distribution enhances the membrane’s photocatalytic effectiveness by maximizing the surface area of ZnO exposed to UV light, thereby facilitating higher rates of ROS production. Additionally, microwave-assisted synthesis allows for rapid, controlled heating, which reduces particle agglomeration and ensures a more homogenous distribution of ZnO-NPs within the membrane. This homogeneity is essential for consistent self-cleaning behavior across the entire membrane surface.

We used the agar disk diffusion method to evaluate the antimicrobial activities of various concentrations. Membrane samples, each measuring 6 mm in diameter, were carefully placed on the surface of nutrient agar in separate Petri dishes. The plates were incubated at 37 °C for 24 h. Following the incubation, the inhibition zones around each membrane were measured to evaluate the antimicrobial activity.

**Figure 17:**
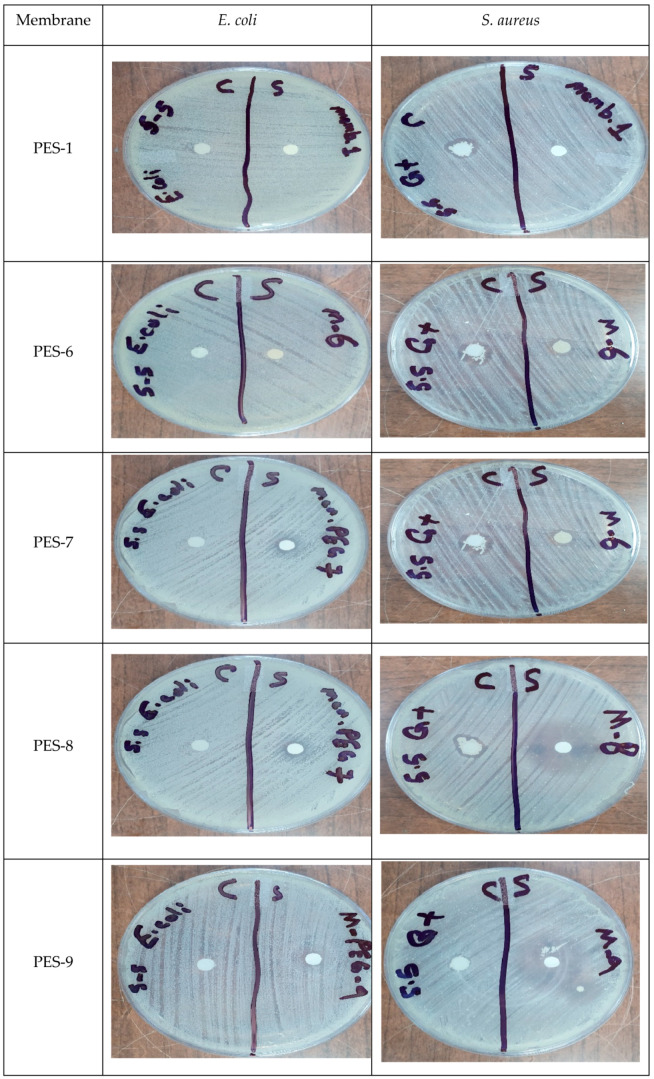
The antibacterial activity of the synthesized PES-ZnO nanocomposite membranes.

**Figure 18:**
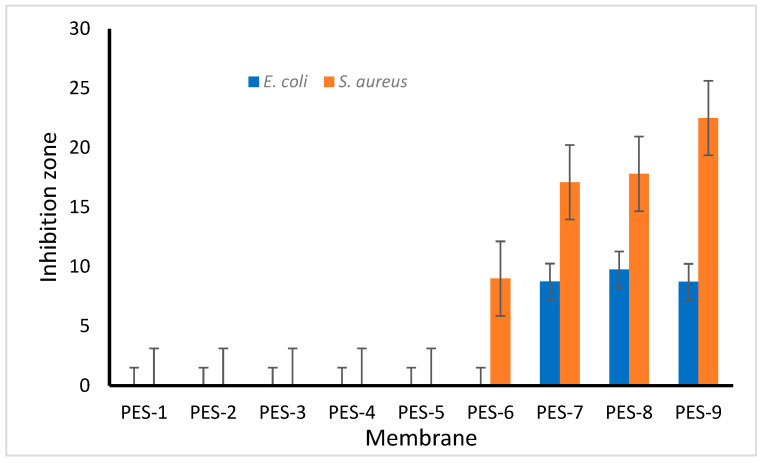
Antibacterial efficacy of PES-ZnO nanocomposite membranes: inhibition zones against Gram-negative and Gram-positive bacteria.

Figure 17 and Figure 18 illustrate the results related to the antibacterial activity of the synthesized PES-ZnO membranes.

The findings demonstrate that the pristine PES membrane, as well as the PES-2 to PES-5 membranes, did not exhibit antibacterial effectiveness against either bacterial strain. Interestingly, the PES-6 membrane, which lacked NaOH and consequently did not contain synthesized ZnO-NPs, showed some unexpected antibacterial activity against *S. aureus* but remained inactive against *E. coli*. The findings further indicate that incorporating ZnO-NPs into the PES membrane significantly enhances antibacterial activity against *S. aureus* compared to *E. coli*. Jo et al. [50] reported similar results. Moreover, the antibacterial efficacy against both bacterial strains increased with higher ZnO concentrations within the membrane matrices.

The antibacterial activity of microwave-assisted, in-situ synthesized polyethersulfone (PES)-ZnO nanocomposite membranes demonstrates promising potential in addressing microbial contamination, largely due to the unique properties of ZnO-NPs. Microwave synthesis facilitates the rapid and uniform formation of ZnO-NPs within the PES matrix, enhancing both the distribution and stability of ZnO. This method allows for better integration of ZnO-NPs, minimizing aggregation and maximizing the exposure of the active ZnO surfaces, which are key to antimicrobial efficacy.

ZnO-NPs exert antibacterial effects through several mechanisms. One key mechanism of action is the production of ROS, including hydroxyl radicals and hydrogen peroxide, which damage bacterial membranes’ cells, proteins, and DNA. This oxidative stress is particularly effective against bacteria such as Escherichia coli (Gram-negative) and Staphylococcus aureus (Gram-positive) by causing cell lysis and death [51]. Additionally, ZnO-NPs can interact with bacterial cell walls through electrostatic forces, compromising membrane integrity and leading to cell leakage. These interactions are enhanced by the high surface area of well-dispersed ZnO-NPs, which improves contact with bacterial cells and increases the antibacterial efficacy of the composite membrane [22].

Overall, the microwave-assisted in-situ synthesis of PES-ZnO nanocomposites offers a controlled and efficient approach to producing antibacterial membranes. The combination of ROS generation [51], membrane disruption, and uniform nanoparticle dispersion enhances the antibacterial properties of the composite, making it an effective solution for environments prone to microbial contamination.

## 4. Conclusions

This study successfully demonstrates the innovative development of polyethersulfone–zinc-oxide (PES-ZnO) nanocomposite membranes using microwave-assisted, in-situ synthesis. By integrating zinc-oxide (ZnO) nanoparticles into the PES matrix, the resulting membranes significantly improve hydrophilicity, permeability, antifouling, self-cleaning, and antibacterial properties. These enhancements are attributed to the synergistic effects of the ZnO-NPs, which improve membrane morphology and surface roughness while generating reactive oxygen species (ROS) that degrade organic contaminants. Incorporating ZnO increases water flux and dye retention efficiency and supports long-term operational stability, making these membranes highly suitable for wastewater treatment applications. The microwave-assisted synthesis method ensures a uniform distribution of nanoparticles and strong integration within the PES matrix, reducing ZnO leaching and enhancing durability. Moreover, the membranes’ ability to resist fouling and mitigate bacterial contamination highlights their potential for industrial-scale applications, particularly in high-salinity and challenging water-treatment circumstances. The findings underscore the importance of advanced material engineering and innovative synthesis techniques in addressing critical environmental challenges, paving the way for scalable and sustainable solutions in water purification and resource recovery.

## Figures and Tables

**Table 1:** Compositions of the ZnO preparations.

	ZnCl_2_ (wt%)	PVP (wt%)	Glucose (wt%)	NaOH (5M) (wt%)	PES (wt%)	DMF (wt%)
NPs-1	10	10	10	0	0	70
NPs-2	10	10	10	0	1	69
NPs-3	10	10	10	0	2	68
NPs-4	10	10	10	0	3	67
NPs-5	10	10	0	0	0	80
NPs-6	10	10	0	10	0	70
NPs-7	10	10	10	10	0	60
NPs-8	10	10	0	10	1	69
NPs-9	10	10	0	10	2	68
NPs-10	10	10	0	10	3	67

**Table 2:** The compositions of the doped solutions.

	PES (wt%)	ZnCl_2_ (wt%)	DMF (wt%)	Glucose (wt%)	PVP (wt%)	NaOH (5M) (wt%)	Microwave Treatment
PES-1	15	0	83	1	1	0	With
PES-2	15	0.1	82.9	1	1	0	With
PES-3	15	0.2	82.8	1	1	0	With
PES-4	15	0.5	82.5	1	1	0	With
PES-5	15	1	82	1	1	0	Without
PES-6	15	1	82	1	1	0	With
PES-7	15	1	82	0	1	1	With
PES-8	15	2	81	0	1	1	With
PES-9	15	5	78	0	1	1	With

**Table 4:** The surface roughness of the PES-ZnO nanocomposite membranes.

Membrane	PES-1	PES-5	PES-6	PES-7	PES-8	PES-9
S_a_ (nm)	108.6	42.3	34.8	52.9	86.7	129.2
S_q_ (nm)	156.3	54.7	46.8	78.9	129.5	178.6

**Table 5:** The water permeability of the PES-ZnO membranes.

	Permeability (L.m^−2^.h^−1^.bar^−1^)	R^2^
PES-1	12.08	0.9957
PES-2	21.84	0.9846
PES-3	32.89	0.9926
PES-4	37.90	0.9959
PES-5	47.57	0.9914
PES-6	63.18	0.9992
PES-7	69.40	0.9877
PES-8	70.28	0.9877
PES-9	76.15	0.9933

## Data Availability

The original contributions presented in this study are included in the article. Further inquiries can be directed to the corresponding author.

## Abbreviations

CA	cellulose acetate
PES	Polyethersulfone
PVDF	polyvinylidene fluoride
PVP	Polyvinylpyrrolidone
BSA	bovine serum albumin
MB	methylene blue
RG	Congo red
DMF	N,N, Dimethylformamide
MF	Microfiltration
UF	Ultrafiltration
NF	Nanofiltration
OR	reverse osmosis
SEM	Scanning Electron Microscopy
AFM	Atomic Force Microscopy
FTIR	Fourier-transform infrared spectroscopy
ZnO	zinc oxide
